# Use of multisensory stimulation in institutionalized older adults with moderate or severe dementia

**DOI:** 10.1590/1980-5764-DN-2021-0022

**Published:** 2022-05-13

**Authors:** Bento Miguel Machado, Carla da Silva Santana Castro

**Affiliations:** 1 Universidade de São Paulo, Programa de Pós Gradução Interunidades em Bioegenharia (EESC/FMRP/IQSC-USP), São Carlos SP, Brazil.; 2 Universidade de São Paulo, Faculdade de Medicina de Ribeirão Preto, Departamento de Ciências da Saúde, Ribeirão Preto SP, Brazil.

**Keywords:** Dementia, Complementary Therapies, Behavior, Health of Institutionalized Elderly, Demência, Terapias Complementares, Comportamento, Saúde do Idoso Institucionalizado

## Abstract

The Multisensory Stimulation Program can help manage behavioral and psychological symptoms of dementia.

**Objective:**

The objective of this study was to investigate the effects of the Multisensory Stimulation Program on behavioral, mood, and biomedical parameters of older adults with moderate and severe dementia compared to a control group not submitted to this program.

**Methods:**

This study is an interventional, parallel, open-label, quasi-experimental clinical trial, which is quantitative and qualitative in nature and is also an exploratory type. The sample was divided for convenience into intervention group (IG) and control group (GC) that did not participate in the Multisensory Stimulation Program. Data analysis included descriptive statistics, nonparametric tests (two-tailed alpha value of 0.1 was applied), and thematic content analysis.

**Results:**

The sample consisted of 20 older adults (IG=10 and GC=10), with a mean age of 83 years, an average of 3 years of education, and moderate or severe dementia. Reduction in intervention group behavioral changes (p=0.059) and numerical improvement in intervention group cognition were observed. A decrease in heart rate (p<0.05) and diastolic blood pressure (p<0.05) was observed before and immediately after the session in the intervention group. The caregivers described engaged behavior in intervention group, while they reported apathetic behavior in control group. Session records described verbal and nonverbal communication and sustained attention for more than 3 min regarding the sensory resource explored.

**Conclusions:**

The Multisensory Stimulation Program could be a new look at the health care practices performed in the nursing homes that consider the older adults’ sensory preferences and may help with dementia behavior management.

## RESUMO.

Um programa de estimulação multissensorial pode auxiliar no manejo dos sintomas comportamentais e psicológicos da demência.

**Objetivo:**

Investigar os efeitos do programa de estimulação multissensorial sobre o comportamento, o humor e parâmetros biomédicos de idosos com demência moderada e grave, quando comparados a um grupo controle não submetido a esse programa.

**Métodos:**

Estudo de intervenção quase experimental, paralelo, mascaramento aberto, de natureza quantitativa e qualitativa, do tipo exploratório. Amostra foi dividida igualmente por conveniência entre grupo de intervenção (GI) e grupo controle (GC), que não participou do programa de estimulação multissensorial. estatística descritiva, testes não paramétricos (valor alfa bicaudal de 10% aplicado) e análise temática de conteúdo.

**Resultados:**

Amostra composta de 20 idosos (GI=10 e GC=10), com média de idade de 83 anos, média de três anos de escolaridade e demência moderada ou grave. Houve redução de alterações comportamentais (p=0.059) e melhora numérica no desempenho da cognição de Grupo de Intervenção. Observou-se diminuição da frequência cardíaca (p<0.05) e da pressão arterial diastólica (p<0.05) antes e imediatamente depois da sessão no grupo de intervenção. Os cuidadores descreveram comportamento engajado no grupo de intervenção, enquanto relataram comportamento apático no grupo controle. Os registros da sessão descreveram ações de interação verbal e não verbal e atenção sustentada por mais de três min diante do recurso sensorial explorado.

**Conclusões:**

O programa de estimulação multissensorial poderia ser um novo olhar sobre as práticas de saúde realizadas em instituições de longa permanência de idosos que considerem as preferências sensoriais do idoso, e pode auxiliar no manejo do comportamento demencial.

## INTRODUCTION

Dementia is characterized by a significant cognitive decline in the performance of cognitive abilities; such deficits interfere with functional capacity^1^. ­Symptoms may vary according to the etiological subtype and stage (i.e., mild, moderate, or severe). The severe stage has a more significant impact on the functional capacity to perform daily activities and results in the loss of sensitive functions, such as hearing and vision^2^ ^,^ ^3^ ^,^ ^4^.

It was estimated that there were about 43.8 million people with dementia in the world in 2016^5^. The incidence of dementia doubles every 6.3 years with increasing age^6^, with an estimated economic cost of 818 billion dollars per year globally^2^. Brazil had about 1.6 million cases with the second-highest prevalence by standardized age (1,037 cases per 100,000 inhabitants) in 2016^5^.

In the severe stage of dementia, pharmacological measures have limited efficacy, complex management, and side effects^7^. Nonpharmacological interventions can effectively manage behavioral and psychological symptoms, especially in institutional contexts^8^, and present lower risks than drug treatment^9^.

The Multisensory Stimulation Program (MSSP) is a nonpharmacological approach in environments that offer sensory experiences. It stimulates the primary senses (e.g., sight, hearing, smell, touch, taste, and vestibular) provided through controlled interventions^10^.

This approach is nondirective. There is no standardization of task sequences without focusing on short-term memory but on momentary experiences^11^. The stimulation usually takes place in a room designed with equipment, such as fiber-optic cables, water columns, aroma diffusers, ambient music, objects with textures, among others^12^.

The MSSP can bring the following benefits to institutionalized older adults with moderate to severe dementia: favor sensory processing abilities and reduce sensory overload^13^; promote improvement in aggressive behavior^14^; increase self-esteem up to 1 week after the last session^15^; have immediate positive effects on mood, behavior, and anxiety^16^ ^,^ ^17^ ^,^ ^18^; and improve communication during morning care between professionals and institutionalized older adults^19^; after the end of the intervention, it can decrease blood pressure (BP)^15^, reduce heart rate (HR) ^15^ ^,^ ^16^ ^,^ ^17^ ^,^ ^18^ ^,^ ^19^ ^,^ ^20^, and increase oxygen saturation^14^ ^,^ ^16^.

No adverse effects were reported in older adults with dementia due to MSSP use. However, moderate investments are needed for its implementation in institutional environments, either in resources, time, or training of caregivers. More research on MSSP with higher methodological quality is also recommended, such as a control group (CG), consistent outcome measures, and generalization of results to other environments^21^ ^,^ ^22^.

There is limited evidence of the benefits of pharmacological interventions on behavioral changes of the older adults with dementia, and physical restrictions are contraindicated^21^. There is also a lack of nonpharmacological intervention programs aimed at this population. Generally, groups of activities with cognitive and motor emphasis are offered to residents, not including the older adults with dementia.

Considering that scientific productions on the subject are incipient, and further studies are recommended, given the scarcity of stimuli in the institutional environment, this study emphasized the relevance of using the MSSP in behavioral changes and parametric biomedical services for institutionalized older adults with moderate and severe dementia. The study methods used a mixed and quantitative approach together with a qualitative approach.

The hypothesis of this study was whether the use of MSSP produces effects in reducing behavioral changes, promoting neutral mood, and producing changes in biometric parameters (e.g., BP and HR). The objective of this study was to investigate the effects of MSSP on behavioral, mood, and biomedical parameters of older adults with moderate and severe dementia compared to a CG not submitted to this program.

## METHODS

### Methodological and ethical issues

This study is an interventional, parallel, open-label, quasi-experimental clinical trial, of mixed nature (quantitative and qualitative), and exploratory type.

This study was approved by the Research Ethics Committee of the Hospital das Clínicas of the Medical School of Ribeirão Preto under opinion no. 11,134/2016. The project was registered in the Brazilian Registry of Clinical Trials (ReBEC): RBR-459x9d. The management of the nursing home approved the collection of information, the installation of the multisensory room, and the performance of interventions. The written informed consent was obtained in the beginning of the study by formal caregivers (e.g., nursing staff). Older adults with dementia were informed about the purpose of this study and verbally agreed to participate.

### Casuistry

A survey of institutionalized older adults diagnosed with dementia was carried out based on the medical records of the nursing home in the Brazilian city of Ribeirão Preto.

Inclusion criteria were as follows: being over 65 years old; having a medical diagnosis of dementia, such as Alzheimer’s dementia, unspecified dementia, or mixed dementia (Alzheimer’s plus vascular/other subtypes); being institutionalized in a nursing home; achieving scores below the cutoff score (<26 points for people with more than 8 years of education, <18 points for 1-7 years of education, and <13 points for illiterate people) according to the Brazilian version of the Mini-Mental State Examination (MMSE) to identify the cognitive status^23^; and having dementia at stage 2 (moderate) or 3 (severe) according to the clinical dementia rating (CDR) to determine the stage of dementia^24^.

The exclusion criteria were as follows: having aphasia of expression or understanding, being confined to bed, and having severe visual impairment or hearing loss that is not corrected by visual and auditory resources.

Formal caregivers (e.g., nursing staff) were invited to fill out standardized instruments and questionnaires about information of the older adults. The inclusion criteria were to work for at least 3 months at the institution before starting the research. The caregivers were selected for convenience: the head nurse suggested which nursing staff was responsible for the primary daily care for each older adult chosen in this study. Due to this type of selection, it was not possible to have blind conditions about the study groups. The family members were not invited because they did not live with the residents in this institution and were sometimes absent.

### Study procedures

#### Sample characterization

It included medical record research (e.g., type of dementia and health comorbidities), questionnaire with sociodemographic aspects (e.g., age, sex, education level, and time living in the institution), and the Katz index to evaluate the functional capacity^25^ (primary daily life activity dependency: mild: 0-1, moderate: 2-4, and severe: 5-6).

Pre-intervention period: Both groups were evaluated with the following standardized instruments before starting the intervention period: Cornell Scale for Depression in Dementia (CSDD) to quantify depressive symptoms^26^ and Neuropsychiatric Inventory (NPI) to quantify behavioral changes in the older adults^27^ ^,^ ^28^. The higher the score obtained, the worse will be the changes in mood and behavior.

A semi-structured checklist was used to collect the perceptions of formal caregivers during the intervention program. Caregivers were asked to select which alternatives were most prevalent in that month regarding behavior (disruptive to engaged), mood (normal to irritable), and level of interaction with the institutional environment and communication with the caregiver (none/very much). There was also an open field to describe notes regarding the behavior and interaction of the older adults.

#### Intervention period

A total of 20 older adults were selected and divided for convenience into intervention group (IG) and CG.

The convenience sample was justified due to the use of the entire sample available during the research period. There were a small number of older adults diagnosed with dementia at the institution, i.e., only 36 of 105 residents. From this small number, participants were excluded due to death and other exclusion criteria. There was no other institution with a multisensory environment in the city. The use of this institution’s sensory room was reserved for its residents.

(A) IG: Notably, 10 older adults received individual care in a multisensory room, twice a week, for 30 min, during 3 months, totaling 24 sessions. Two researchers participated in the session, of whom one performing the interventions and the other one, as an observer, recording the reactions and data of the intervention. The IG participants continued to attend the activities promoted by the institution’s health care team.

A semi-structured script for observation adapted and modified from the preliminary version of the Snoezelen Assessment Scale^29^ was used during the sessions to describe the subjects’ reactions to the stimuli presented during the sensory stimulation intervention and to record the sensory resource used and time spent in it. The therapist measured the systolic blood pressure (SBP), diastolic blood pressure (DBP), and HR right after the older adult arrived at the environment and the end of the session, using an automatic digital BP monitor.

(B) CG: CG was formed with 10 older adults who did not participate in the MSSP but continued to participate in activities promoted by the institution’s health care team.

Post-intervention period: Both groups were reevaluated with instruments of the pre-intervention period after the end of the therapeutic program (3 months).

#### Session structure

The MSSP consisted of a set of sessions based on the multisensory stimulation concept^11^ ^,^ ^30^. The therapist’s approach is nondirective. In the first session, therapist invites the participant to explore the spaces, introducing each resource one at a time, and observes the participant’s reactions (verbal and nonverbal). Positive responses allow the therapist to again show the most preferred stimuli in future sessions. Adverse reactions, i.e., repulsion to specific resources, can be avoided. Verbal or nonverbal positive reinforcements can be prepared from the participant’s responses to the stimuli as a form of positive feedback, favoring a sense of effectiveness for making such a choice^30^.

In the following sessions, the therapist can prepare the environment in advance with some resources that obtained favorable responses, based on observing the participant’s reactions, allowing the preparation of a sensory diet (personalized program)^31^ and prioritizing the most interesting stimuli. Based on the therapist’s observations, some more excitatory sensory stimuli could be offered for those with more apathetic and depressed behavior, and more relaxing stimuli could be offered for those with more agitated, anxious, or irritable behavior.

The participant must be able to have accessible handling, easy exploration, and visual recognition of the resources, which integrates the primary senses^31^ ^,^ ^32^ ^,^ ^33^. The therapist could use fewer verbal commands, allowing the participant to explore the materials and encourage decision-making^30^.

The participants can spend as much time as they want in the space/resource, even if they wish to stay there for the entire session. The session ended at 30 min or when the older adult showed signs of tiredness or restlessness that were not mitigated by the stimuli.

Three environments were created at the institution based on friendly design principles for people with dementia. Some principles such as being a safe, familiar, and visually accessible environment; favoring engagement in daily activities; and optimizing good stimulation^32^.

**Figure 1.**
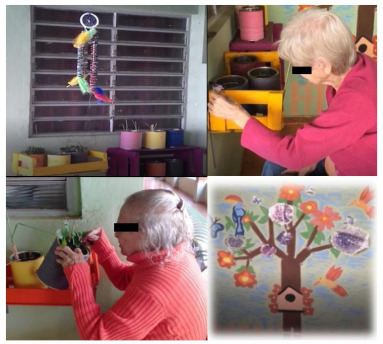
The sensory garden had aromatic plants, wind bell, and textures in painting on the wall.

**Figure 2.**
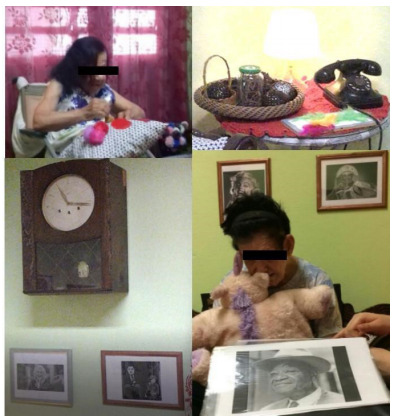
The vintage room had decoration features from the 1940s to 1960s, a photo album, flavored sachets and topiaries, tactile pillows, feather fan, stuffed animal, thermal bag and hand massager, ambient music, and a tambourine.

**Figure 3.**
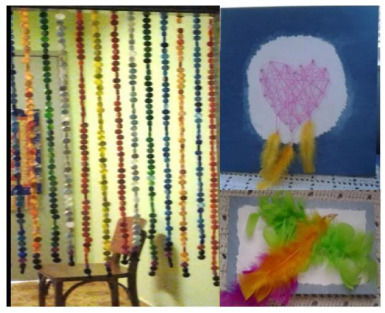
The transition corridor (between the vintage room and the lights room) had a beaded curtain and pictures with different textures on the wall.

**Figure 4.**
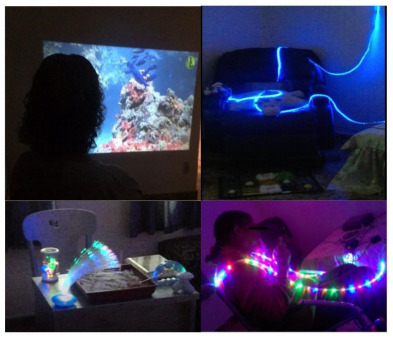
The lights room had optical fiber light, LED lights on the roof, overhead projector, aroma diffuser, and ambient music.

The spatial order was sensory garden (e.g., plants with different aromas) (Figure 1), vintage room (e.g., decorative elements from the 1940s to 1960s) ­(Figure 2), transition corridor (e.g., beaded curtain and pictures with different textures) (Figure 3), and finally, the lights room (e.g., colorful lights, music, and olfactory elements) (Figure 4). The places were similar to a domestic space, facilitating the transition of the older adult between physical spaces^33^. Examples of low-cost sensory resources were illustrated in Figures 1-4, of which many were handcrafted or purchased at stationery, party, and craft stores.

The following is an example of a session used in this study based on these references^11^ ^,^ ^30^ ^,^ ^31^ ^,^ ^33^.

“The session started in the garden. The participant handled the dream filter and commented on what she saw in that space (flowers, drawing on the wall) for about 5 min. The therapist asked if she wanted to stay there or move to the vintage room. In the vintage room, she sang the ambient music for a few minutes; then she stopped singing when she saw a stuffed toy on a chair. She hugged and caressed the toy, asked questions about it, exclaimed that it is so cute (...). After some minutes, she spontaneously described what she saw in a painting on the wall (...). In the lights room, she pointed with her index finger to the video of birds in nature, flexed her back, and yelled, “it is looking at me!” - with a surprised facial expression and laughing a lot. She talked to the therapist about the videos for some time (...). When the time ended (30 min), she was informed that they should go back to the living room.”

#### Data analysis

Statistical analysis was performed using the Minitab version 19 software. The Ryan-Joiner test was used to determine whether or not the data followed a normal distribution. Variance coefficient and skewness (asymmetry) values were also observed. The Bonett’s and Levene’s tests were calculated to assume variance equality between variables (by groups and between groups). The Grubbs’ test was used to detect outliers. A two-tailed alpha value of 0.1 was applied. The Mann-Whitney nonparametric U-test was used to verify whether there were statistical differences between groups (independent variables). The Wilcoxon signed-rank nonparametric test was used to verify the before and after measures by group (dependent variables).

Thematic content analysis was used for qualitative analysis of the data from the observation records of the sessions carried out by the following three chronological steps^34^ ^,^ ^35^.

#### Pre-analysis

Organization of observation records for each session to facilitate and systematize initial ideas.

#### Material exploration

Exhaustive reading, classification, and categorization of themes and creation of thematic units that appeared more frequently in the records. The thematic units were built from the actions observed by the participants in the multisensory environment, based on the following themes: behavior, mood, interaction, and cognition.

#### Treatment of results

Analysis of thematic units, allowing the authors to propose inferences and interpret the data obtained according to their theoretical framework and the objectives foreseen in the research^34^ ^,^ ^35^.

## RESULTS

### Characterization of the participants

There were 105 residents in the nursing home. The residents were excluded from this study due to the following exclusion criteria: 69 older adults without a dementia diagnosis and 16 with dementia, but 5 were confined to the bed, 4 with aphasia expression, 3 with mild dementia, 2 were visually impaired, and 2 died before the sensory room was completed.

The sample consisted of 20 older adults with a mean age of 83 years, with 17 women and 3 men with an average of 3 years of education, diagnosed with moderate or severe dementia and institutionalized in the nursing home for about 4 years, partially dependent on self-care (Table 1). Most of the older adults presented socioeconomic vulnerability, weakened family ties, and came from rural areas.

**Table 1.** Characterization of the intervention group and control group regarding age, sex, education level, time in the institution (in years), types, and stages of dementia, comorbidities, and functionality.

Categories		IG	CG	Total
		n=10	n=10	n=20
Age	Mean	84.4	80.7	82.7
	SD	6.8	8.5	7.7
Sex	Female	10	7	17
	Male	0	3	3
Education level	0 years	4	1	5
	1-4 years	5	8	13
	Over 8 years	1	1	2
Time in the institution	Up to 1 year	3	2	5
	1-4 years	4	4	8
	Over 4 years	3	4	7
Types of dementia	NS	6	8	14
	Mixed	3	1	4
	Alzheimer	1	1	2
Stages of dementia	Moderate	4	2	6
	Severe	6	8	14
Comorbidities	Depression	3	0	3
	Parkinson	1	1	2
	Prior stroke	1	1	2
	COPD	1	2	3
Dependence on BADL	0-1	1	3	4
	2-4	7	3	10
	5-6	2	4	6

IG: intervention group; CG: control group; SD: standard deviation; NS: not specified; COPD: chronic obstructive pulmonary disease; BADL: basic activities of daily living.

Notably, 11 formal female caregivers were consulted, including 7 nursing technicians, 2 nursing assistants, and 2 caregivers (training course).

### Effects on behavior, mood, and cognition in the institutional environment

The MSSP decreased the behavioral changes (p=0.059) of the IG during the intervention period. There was no statistically significant difference when the groups were compared to each other (Table 2).

**Table 2.** Effects on behavior, changes in mood, and cognitive capacity of both groups in the pre- and post-intervention period.

		IG		CG	
Behavior (NPI)	Score: (0-144 points)	Pre	Post	Pre	Post
	Mean	43.3	28	32.5	29.8
	Median	41.5	18.5	28	25.5
	Standard deviation	25.9	31	20.1	17.9
	Pre and post by groups*	p=0.059		p=0.918	
	Between groups*	p=0.161			
Mood (CSDD)	Score: (0-38 points)	Pre	Post	Pre	Post
	Mean	11.1	9.4	10.3	8
	Median	9.5	9.5	11	7
	Standard deviation	7.1	6.1	4.2	4
	Pre and post by groups	p=0.406		p=0.235	
	Between groups	p=0.97			
Cognition (MMSE test)	Score: (0-30 points)	Pre	Post	Pre	Post
	Mean	11.1	12.2	6.8	5.8
	Median	11.5	14	7.5	4.5
	Standard deviation	3.2	3.8	4.6	5.4
	Pre and post by groups	p=0.153		p=0.441	
	Between groups	p=0.109			

IG: intervention group; CG: control group; NPI: Neuropsychiatric Inventory; CSDD: Cornell Scale for Depression in Dementia; MMSE: Mini-Mental State Examination. *Two-tailed alpha value of 0.1 (p<0.1).

Both groups showed a numerical improvement in mood symptoms, with no statistically significant difference between the pre- and post-intervention measurements and in relation to the groups compared to each other (Table 2).

The IG showed a numerical improvement in cognitive capacity, while the CG had a numerical worsening. There was no statistically significant difference between the pre- and post-intervention measurements and when the groups were compared to each other (Table 2).

The caregivers had the perception that the IG had more engaged behavior (nondisruptive, collaborative, and interactive behavior) in the institutional environment over 3 months. The caregivers reported that the IG seemed to interact better with the institutional environment upon returning from the multisensory environment after the sessions. For example, these participants showed a happy facial expression, gaze directed to the physical environment around them, were more communicative with caregivers, and had better interaction among the residents. The caregivers described that the CG had less engaged behavior and more apathetic behavior in the institutional environment over 3 months. For example, these participants reduced their conversations with caregivers, and they worsened the quality of dialogues (e.g., negative verbalizations, shouting, and increased vocal volume). In some cases, adverse reactions occurred during self-care (e.g., moaning, nodding, and closed facial expressions).

### Effects on behavioral, mood, cognitive, and biomedical parameters in the multisensory environment

The session records described beneficial effects in the IG during the session in the multisensory environment. A summary of the central thematic units of content analysis was described (Table 3).

**Table 3.** Intervention group behaviors, mood, and interaction observed in the multisensory environment.

**Behavior**	**Thematic units**	**Observed actions**
	Decrease in apathy	Greater resources exploration, whether requested by a therapist or on their initiative.
	Relaxation response	Naps, slow movements, and relaxed posture.
	Excitatory response	Body and dance movements, smiles, and waves of laughter.
Mood	Euthymic mood	Feeling of well-being.
	Adverse effects on mood.	They were not observed.
	Labile or irritable mood	It was rare, softened with the course of the session.
	Promotion of volition	As the participants knew the environment, they requested more materials of their preferences. Resource exploration and decision-making initiatives without therapist’s interference.
Interaction	Verbal communication	Verbalization of preferences and interests.
	Nonverbal communication	Reactions and facial expression, body language, eye contact, and sensory exploration of objects.
	Stuffed animals	When entering the room, visual search for the object, change in the timbre of the voice and serene facial expression, and presence of hugs and caresses to the thing.
	Ambient music	Recognition of sounds, closing of the eyes, singing pieces of music, dance movements, clapping and foot tapping along with the musical rhythm, and storytelling of life.
Cognition	Cognitive abilities	Stimulation of long-term memory and sensory perception. Sustained attention for more than 3 min in specific resources of the participant’s interest, especially tactile and visual ones, which were usually offered in the foreground.

A higher frequency of engaged behavior and decreased apathy was observed during the session. Participants showed the signs of relaxation or excitement according to the types of stimuli offered. The communication was described by verbal and nonverbal actions, directed at the therapist, stuffed animal, or ambient music. A neutral mood with a sense of well-being and volition was reported. A favoring of cognitive abilities was observed, such as sustaining attention for more than 3 min, long-term memory, and sensory perception.

The SBP, DBP, and HR indicated decreased measurements at the end of the session. A significant difference was found in the pre- and post-intervention measurements of DBP (p<0.05) and HR (p<0.05) attested by the Wilcoxon signed-rank nonparametric test. There was a numerical improvement in the SBP measurements but no significant difference (Table 4).

**Table 4.** Effects on biomedical parameters of intervention group in the pre- and post-session period at multisensory environments.

Biomedical parameters		Pre	Post
SBP (mmHg)	Mean	12.85	12.65
	Median	13	12.55
	Standard deviation	2.05	1.79
	Pre and post	p=0.177	
DBP (mmHg)	Mean	7.62	7.18
	Median	7	6.8
	Standard deviation	2.04	1.84
	Pre and post	p<0.01	
HR (bpm)	Mean	80.76	78.72
	Median	80	80
	Standard deviation	12.48	11.99
	Pre and post	p<0.01*	

SBP: systolic blood pressure; DBP: diastolic blood pressure; HR: heart rate. *Two-tailed alpha value of 0.1 (p<0.1).

## DISCUSSION

This study found the positive effects of MSSP in older adults with moderate and severe dementia on behavioral, interactive, and biomedical parameters in the multisensory and institutional environment compared to a CG not submitted to the interventions of this program.

The MSSP can affect the behavior of older adults with dementia in nursing homes based on the reduction of behavioral changes, such as reduced agitation and apathetic behavior^16^ ^,^ ^18^ ^,^ ^30^. Positive emotions increase the interaction with the environment, redirecting to engaged behavior^36^.

The IG’s engaged behavior observed by caregivers and described in the session records may be related to the therapeutic process within the multisensory environment that allowed the possibility of choices, favoring an engaged, interactive, and collaborative behavior. ­Given the participants’ stage of dementia, the interventions demonstrated a rich potential to stimulate cognitive and social abilities.

The enhancement of the stimulus in multisensory environments can also assist in a balanced sensory processing^37^, by controlling the number of competing stimuli and the intensity of the stimulation, combining sensory preferences and individual needs^13^.

The nursing home surveyed had few opportunities for the older adult with severe dementia to receive a personalized and continuous approach. A program more focused on the needs of the most committed older adults, even if performed collectively or in small groups, would be more appropriate.

In this study, the quantitative improvement in mood symptoms in both groups could be related to the interfering variables since both groups had drug changes in this period, being more present in the CG, according to the medical record and caregivers’ reports.

Positive interaction reactions were described in this study. Such data draw attention to the importance of favoring an enriched routine in nursing homes through a more significant offer of structured and meaningful activities to the older adults with dementia. Other studies have found an improvement in communication and interaction with the environment after multisensory interventions^14^ ^,^ ^38^.

Another interesting fact in this study was the interaction between the older adults and stuffed animals. This resource could help older adults with moderate or severe dementia express unmet needs^39^ and can be a behavioral change strategy^40^.

More humanized actions within the nursing home should consider the identification of elements that influence the interaction. It should also consider which barriers can be mitigated through the design of environments to achieve higher levels of social inclusion^41^. Optimizing the physical environment is essential to facilitate participation in daily activities and help the older adult feel at home^42^.

The effects of MSSP on the cognitive status of older adults with moderate or severe dementia have been poorly studied^22^; Baker et al.^43^ found no change in the average cognitive performance.

The IG’s numerical improvement of cognitive performance and sustained attention described during the sessions (Table 3) may also be related to the person-centered therapeutic process developed in a multisensory approach, having structured sessions with targeted stimuli composed of attractive stimuli elements that arouse the older adults’ curiosity. The worsening in GC’s cognitive performance may be related to the progression of dementia associated with the few stimuli offered in the institutional environment to the population of this study.

Studies that have analyzed the relaxing effect of MSSP on biomedical parameters have not provided conclusive data on its effectiveness^14^ ^,^ ^15^ ^,^ ^20^. In this study, there were methodological differences regarding these studies. One possibility for the variation in BP and HR may be related to the less agitated state of the participants or to the fact that they are more adapted to the environment throughout the session. Interference factors must be considered, such as variability between subjects. The MSSP offered relaxing or exciting stimuli according to the degree of interest of the older adults in the resources offered. Thus, part of the sessions showed an increase in the measures described.

### Study limitations and recommendations for future research

Some factors that could be considered interference variables were as follows: changing medications, dynamics of the institution (changing the older adults’ rooms and changing institutional routine), and high turnover of the nursing staff. The CG had more participants with medication changes (replacing, increasing, or changing the dosage), hospital admissions, and complications (agitation, confusion, or irritability, requiring tranquilizers on this day) compared to the IG.

There were difficulties in recruiting eligible older adults. The sample size may have influenced the ability to determine statistical significance in the quantitative variables. Larger samples, identification and optimum monitoring of interfering variables, and long-term longitudinal studies are suggested for future research.

There was a lack of information in the institution regarding factors that interfered with the behavior of older adults with dementia. Institutions with a staff-to-resident ratio balance would better observe the older adults’ routine, better care management, and more reliable and qualified research interlocutors.

Regarding biomedical data measurements, relaxing or exciting sessions should be analyzed separately since, in theory, they would have different measurement trends, causing statistical interference in the data. These measurements could also be compared with a CG to serve as a standard reference to these variables.

This study identified a higher quality of observations regarding the reactions and behaviors generated during the interventions using a mixed approach (quantitative and qualitative), differing from the studies already carried out with only the quantitative approach.

The low cost of implementing a multisensory room with the characteristics described here, covering older adults with low education and with moderate and severe stages of dementia, is a viable possibility to be reproduced in other institutions.

Finally, it proposes a new look at the health care practices performed in nursing homes that consider the individuality of the older adults with dementia and their sensory preferences and interests, stimulating their participation in collective contexts and supporting their demands.
